# Complete Genome Sequence of Phormidium yuhuli AB48, Isolated from an Industrial Photobioreactor Environment

**DOI:** 10.1128/mra.00759-22

**Published:** 2023-01-10

**Authors:** Yilin Qiu, Avery J. C. Noonan, Kalen Dofher, Moritz Koch, Brandon Kieft, Xuan Lin, Ryan M. Ziels, Steven J. Hallam

**Affiliations:** a Genome Science and Technology Program, University of British Columbia, Vancouver, British Columbia, Canada; b ECOSCOPE Training Program, University of British Columbia, Vancouver, British Columbia, Canada; c Department of Microbiology and Immunology, University of British Columbia, Vancouver, British Columbia, Canada; d Graduate Program in Bioinformatics, University of British Columbia, Vancouver, British Columbia, Canada; e Department of Civil Engineering, University of British Columbia, Vancouver, British Columbia, Canada; f Bradshaw Research Institute for Minerals and Mining, University of British Columbia, Vancouver, British Columbia, Canada; g Life Sciences Institute, University of British Columbia, Vancouver, British Columbia, Canada; University of Southern California

## Abstract

We report the genome of Phormidium yuhuli AB48, which includes a circular chromosome and a circular plasmid (4,747,469 bp and 51,599 bp, respectively). This is currently the only closed reference genome of an isolate of the *Phormidium* genus, based on the Genome Taxonomy Database (GTDB), providing a potential model system for sustainable biotechnology innovation.

## ANNOUNCEMENT

Cyanobacteria have the potential to be harnessed as climate-positive microbial cell factories. Therefore, closed reference genomes for cyanobacterial isolates and mobile genetic elements are needed to provide biological parts for phylogenetic reconstruction, metabolic modeling, and pathway engineering. Members of the *Phormidium* genus, which are capable of forming dense biofilms, have recently emerged as candidate cyanobacterial chassis to realize this potential (1–3).

Here, we report the complete genome sequence of Phormidium yuhuli AB48, which was first described by Koch and colleagues (4). P. yuhuli AB48 was isolated from an industrial photobioreactor environment operated under elevated temperature (35°C to 45°C), salinity (10 g/L), and alkalinity (pH 9 to pH 11). Grown as a biofilm under these conditions, AB48 was found to co-occur with several other microorganisms (5) prior to isolation using a gradient culture method (4). To prepare genomic DNA for sequencing, the isolate was cultured under continuous light in 50 mL of Zarrouk medium (6) in an Erlenmeyer flask for 96 h prior to biomass collection. Genomic DNA extracted using a cetyltrimethylammonium bromide (CTAB)-chloroform extraction protocol (7) was sequenced on both Illumina HiSeq and Oxford Nanopore Technologies (ONT) MinION platforms. Default parameters were used for software related to sequencing data processing and analysis, unless otherwise noted.

For short-read sequencing, the NEBNext Ultra DNA library preparation kit (New England Biolabs) was used. DNA libraries were sequenced on the Illumina HiSeq platform, and 283,172,660 paired-end 150-bp reads (42,475.9 Mbp) were subsampled to 5,659,080 reads (848.9 Mbp) by BBMap (v.38.18) prior to assembly, for an average coverage of 170×. Trimming and quality filtering were performed using BBDuk (v.38.93), which removed 2,818 reads (4.3 Mbp). For long-read sequencing, size selection was performed using the Circulomics short-read eliminator XS kit (Pacific Biosciences [PacBio]) to enrich for fragments longer than 10 kbp. The sequencing library was prepared using the NEBNext companion module for ONT ligation sequencing (New England Biolabs), ONT ligation sequencing kit (SQK-LSK109), and Flongle sequencing expansion kit (EXP-FSE001) and then was sequenced with a Flongle flow cell (FLO-FLG001) on the MinION Mk1b sequencer. Base calling was performed using Guppy (v.5.0.16) and resulted in 130,887 ONT reads (834.3 Mbp), with an *N*_50_ value of 9,790 bp. A total of 64,617 reads (410.8 Mbp) passed quality filtering (Q values of >10 [*N*_50_, 9,600 bp]), for an average coverage of 175.97×. Adapters were trimmed using Porechop (v.0.3.2) (8). Resulting short- and long-read data were hybrid assembled using Unicycler (v.0.4.8) (9). Two circular contigs, of 4,747,469 bp (GC content, 51.68%) and 51,599 bp (GC content, 48.61%), were assembled, corresponding to the isolated cyanobacterial genome and its associated plasmid, respectively, based on contig circularization prediction with Unicycler and classification using Plasflow (v.1.1) (9, 10).

Phylogenetic classification using GTDB-Tk (v.0.3.2) (11) indicated that the isolate is a new member of the *Phormidium_A* genus. The isolate genome was compared to all 12 isolate genomes and metagenome-assembled genomes (MAGs) within this Genome Taxonomy Database (GTDB)-defined genus on the basis of average nucleotide identity (ANI), using FastANI (v.1.32) (12). The genome of Sodalinema gerasimenkoae sp. strain IPPAS B-353 (13) (identity, 87.3%) was the most similar. Koch et al. designated this isolate a new species in the genus *Phormidium*, with the name Phormidium yuhuli AB48 (4). Open reading frame (ORF) prediction and genome annotation for Phormidium yuhuli AB48 were performed using the NCBI Prokaryotic Genome Annotation Pipeline (PGAP) (v.6.2) (14). The complete genome contains 4,187 genes, 4,133 coding sequences (CDSs), 6 rRNA genes (two sets of 5S, 16S, and 23S rRNA genes, consistent with other members of the genus [1]), 3 ncRNAs, and 45 tRNA genes.

### Data availability.

Data from this project are publicly accessible through the NCBI under the BioProject accession number PRJNA834472 and the BioSample accession number SAMN28044976. Raw sequencing data are available from the Sequence Read Archive (SRA) under the SRA accession numbers SRX15625011 and SRX15625009. This BioProject also includes BioSample accession number SAMN28044958; this represents a distinct sample and contains a PacBio data set and metagenome assembly from the photobioreactor enrichment from which P. yuhuli AB48 was isolated (5).
